# A Novel Method for Training the Interdiction of Restricted and Hazardous Biological Materials by Detection Dogs

**DOI:** 10.3389/fmed.2022.847620

**Published:** 2022-04-12

**Authors:** Melissa Singletary, Sarah Krichbaum, Thomas Passler, Lucia Lazarowski, Terrence Fischer, Scott Silvis, L. Paul Waggoner, Paul Walz, Craig Angle

**Affiliations:** ^1^Canine Performance Sciences Program, College of Veterinary Medicine, Auburn University, Auburn, AL, United States; ^2^Department of Anatomy, Physiology, and Pharmacology, College of Veterinary Medicine, Auburn University, Auburn, AL, United States; ^3^Department of Clinical Sciences, College of Veterinary Medicine, Auburn University, Auburn, AL, United States; ^4^Department of Pathobiology, College of Veterinary Medicine, Auburn University, Auburn, AL, United States

**Keywords:** detection dog, bio-agent detection, viral detection, canine, bio-detection, bio-threat detection

## Abstract

The interdiction of restricted and hazardous biological agents presents challenges for any detection method due to the inherent complexity of sample type and accessibility. Detection capabilities for this category of agents are limited and restricted in their mobility, adaptability and efficiency. The potential for identifying biological agents through a volatile organic compound (VOC) signature presents an opportunity to use detection dogs in a real-time mobile capacity for surveillance and screening strategies. However, the safe handling and access to the materials needed for training detection dogs on restricted or hazardous biological agents prevents its broader application in this field. This study evaluated the use of a polymer-based training aid in a viral detection model using bovine viral diarrhea virus mimicking biosafety level 3+ agent conditions. After the biological agent-based odor was absorbed into the polymer, the aid was rendered safe for handling through a rigorous sterilization process. The viral culture-based training aid was then used to train a cohort of detection dogs (*n* = 6) to discriminate agent-based target odor in culture from relevant distractor odors including non-target biological agent-based odors. Following culture-based training, dogs were tested for generalization to aids with infected animal sample-based odors across five sample types (fecal, blood, nasal, saliva, and urine). Within the context of the polymer-based training aid system, dogs were successfully trained to detect and discriminate a representative biological viral agent-based odor from distractor odors with a 97.22% (±2.78) sensitivity and 97.11% (±1.94) specificity. Generalization from the agent-based odor to sample-based odors ranged from 65.40% (±8.98) to 91.90 % (±6.15) sensitivity and 88.61% (±1.46) to 96.00% (±0.89) specificity across the sample types. The restrictive nature for mimicking the access and handling of a BSL 3+ agent presented challenges that required a strict study design uncommon to standard detection dog training and odor presentation. This study demonstrates the need to further evaluate the utility and challenges of training detection dogs to alert to biological samples using safe and manageable training aids.

## Introduction

Biological targets of interest represent a category of complex and relatively inaccessible threats for which instrumentation and traditional test methods are limited in rapid adaptation, mobility, and deployability (1). Biological threats can be manufactured and deliberately dispersed or occur through natural outbreaks and spread rapidly without being detected in real-time. The first line of defense in the detection of biological targets necessitates a rapid mobile technology to direct support resources, such as law enforcement, security teams, public health professionals and laboratories, toward suspect areas, materials and/or individuals. Programs, such as those outlined in the recent 2021 National Blueprint for Biodefense, do not readily have the capability to detect biological agents in real-time and state “More than 5 years after we released A National Blueprint for Biodefense [2015], the United States remains at catastrophic biological risk,” indicating a critical security gap (2). This was echoed in the 2021 Global Health Security Index stating, “…all countries remain dangerously unprepared for meeting future epidemic and pandemic threats” and cite real-time surveillance as a capacity of potential international concern (3). The 2015 World Organization for Animal Health (OIE) Biological threat reduction strategy where surveillance, early detection and rapid response of bio threats was identified as one of the most sustainable and effective means of protection outlined in their strategy (4). The real-time detection of biological agents would provide governments with instant intelligence that could prevent, allow for early interdiction and intervention in, or confine a biological threat through precision resource allocation.

Detection dogs are a valuable threat detection asset used across disciplines from traditional law enforcement targets, such as explosives and narcotics, to novel applications in medical and biological detection (5–9). In a previous study for viral detection using trained detection dogs, our group demonstrated a detection capability for bovine viral diarrhea virus (BVDV) in live culture and successful discrimination of that virus from similar viruses, i.e., bovine herpes virus (BoHV-1) and bovine parainfluenza virus 3, in live culture (10). This model for virus detection represents a biosafety level 2 (BSL2) agent that affects multiple species and has closely related viruses of foreign animal disease significance. BVDV is in the *Pestivirus* genus alongside classical swine fever virus and border disease virus and belongs to the *Flaviviridae* family which encompasses viruses of zoonotic concern such as yellow fever virus, Zika virus, Dengue virus, and West Nile virus (11, 12). It is reported that at least three-quarters of human emerging infectious diseases originate in animals and four-fifths of potential biothreat agents are zoonotic, meaning they can be transmitted from animals to humans (4). The use of this BVDV model as a known canine detection capability for use in this study to mimic a restricted and hazardous agent in the development of a canine training aid provides a robust means for proof of concept under operationally relevant conditions.

Accessibility and technical proficiency required with sample handling for biological agents of high significance, especially BSL 3+, limit the feasibility of applying traditional training techniques toward restricted and hazardous biological targets of detection. The biosecurity levels represent the associated categorization of risk and increasingly restrictive standards for access and handling. A BSL-2 agent is considered by the Centers for Disease Control (CDC) to represent a human-associated disease agent that poses a moderate level of hazard to the handling personnel and/or to the environment, including animals and requires special practices of limited access and containment measures (13). Advancement to BSL-3 classified agents represent indigenous or exotic agents that can result in serious or potentially lethal disease and requires severely restricted access to designated and approved facilities, qualified personnel and multiple containment measures (14). Alternative training materials that represent select chemical components of larger target odor profile have been used in other disciplines with detection dogs to overcome the limitation of access to hazardous or restricted materials but establishing a biological agent-based odor profile with current instrumentation sensitivities remains a challenge (15). Additionally, identifying peak compounds does not necessarily represent the relevant odor profile for canine learning and biological agent recognition as these are complex odor signatures and a combination of signals is likely more representative of a unique odor profile rather than a single isolated compound. The field of volatile compound analysis has expanded and made significant gains toward higher sensitivity. Odors are predominantly comprised of volatile organic compounds (VOCs) which represent a category of low molecular weight compounds that are volatile under normal conditions (16, 17). However, complex targets remain difficult to identify with a unique odor “fingerprint” and they are dynamic samples that can change over time (18). Therefore, selection of an easily reproducible primary odor target for use as a pseudo-training aid, which does not use the original true material for its production, presents a challenge and may be limited in operational relevance (19).

Recent studies with a polymer-based odor capture and release (POCR) training aid demonstrated its capability of presenting qualitatively the same target-based odor profile for explosives such as triacetone triperoxide (TATP), for use in detection canine training (20–22). This aid represents a non-pseudo alternative that uses the true material in its manufacture directed toward ad/bsorption of the full target odor profile (19) while eliminating the associated risks and hazardous of handling and use. This technology uses a polymer-based material to safety capture the odor profile of a target of interest, which holds application toward biological targets with complex odor signatures. This technology provides an option for the safe presentation of the captured odor to dogs for use in training. The nature of the polymer material suggests it can physically withstand sterilization. This is a critical step needed in a potential training aid against biological threats as it mitigates the associated risk of exposure or contamination to biological targets while concurrently maintaining an ability to access and handle the odor outside of a laboratory setting for use in training with detection dogs.

This study aims to evaluate the use of the POCR training aid technology with hazardous biological agents under BSL 3+ conditions with the model BVDV virus. Within the POCR training aid system, dogs were trained to discriminate BVDV culture-based odors from relevant distractor odors and other non-target viral agent-based odors using the POCR training aid and were tested for generalization to POCR aids with infected animal sample-based odor across five sample types (fecal, blood, nasal, saliva, and urine) as a potential restricted and hazardous agent capability. We hypothesized that using the odor ad/bsorption strategies for the POCR training aid technology and sterilization procedures to mimic a BSL 3+ biological threat would provide proof-of-concept for a safe odor presentation method in canine detection training with restricted and hazardous biological materials.

## Materials and Methods

All activities were approved and monitored by the AUCVM Institutional Animal Care and Use Committee (IACUC #2019-3514). The AUCVM is an Association for Assessment and Accreditation of Laboratory Animal Care International (AAALAC) accredited facility and Biological Use Authorization was granted by the Auburn University Institutional Biosafety Committee.

### Cattle

A prospective study was designed to control for confounding variables present in naturally occurring disease. Self-controls (animal samples collected prior to infection) and the full spectrum of infectious disease course from incubation period to recovery were available for sampling. Thirty, approximately 12-month-old, 800 lbs (365 kg) steer calves obtained from Animal Health Research (AHR), Auburn University, were utilized in this study and maintained in isolated pastures at the North Auburn BVDV unit. Each pasture has a dedicated corral and covered work area with chute. Diet and husbandry were identical between the two groups. Virus isolation and antibody screening assays were performed on the calves that were available to use in the study. All cattle were negative on virus isolation for BVDV and seronegative to BoHV-1, and all BVDV group cattle were confirmed seronegative for BVDV.

Group 1 cattle (BVDV-1; *n* = 20) were housed in a pasture that was separated from the pasture housing Group 2 cattle (BoHV-1, *n* = 10) by at least 9 m. Cattle were acclimated to the pastures for 3 days, followed by collection of samples as described below beginning on day −5. On day 0, each animal was infected with either 5 mL of BVDV inoculum containing 10^6^ cell culture infective dose 50% (CCID_50_) of BVDV-1b AU526 per ml (Group 1) or 5 ml of BoHV-1-1 Colorado (Cooper) strain containing 1 x 10^7^ CCID_50_ per ml (Group 2). Viruses were propagated under identical conditions in minimal essential medium (MEM) with Earle's salts, containing equine serum, L-glutamine, sodium bicarbonate, penicillin/ streptomycin/amphotericin (PSF) and purified water. All cattle were inoculated by intranasal instillation using 1-inch plastic intranasal catheter tips attached to a 5 mL single-use syringe.

Samples were collected from each steer on days −5, −4, −3, −2, −1, 0, 1, 2, 3, 4, 5, 6, 7, 8, 9, 10, 14, 21, and 28. Sample types collected on each sampling day included: blood, nasal swabs, salivary swabs, urine, and feces. For sample collection, cattle were restrained in a squeeze chute. Blood samples were collected for canine training and surveillance of viral infection by virus isolation. Blood collection was performed by venipuncture from the jugular vein utilizing a vacuum tube system consisting of a Sarstedt Monovette® tube, Sarstedt needle adapter, and an 18-gauge, 1.5-inch needle. A total of 25 ml of blood was collected from each animal in serum separator tubes and in tubes containing EDTA for eventual isolation of white blood cells. Nasal and salivary samples were collected by swabbing each nostril and mouth with separate sterile cotton-flocked swabs of approximate 0.69” tip length size. Samples were placed directly into sterile cryovials. Fecal samples (~20 grams) were manually collected by inserting a gloved hand or fingers into the rectum, and then samples were placed into empty plastic cryovial containers. Additionally, urine was collected into sterile urine collection cups, when possible, either when the animal urinated voluntarily while in the chute or following gentle stimulation (“feathering”) of the prepuce, then transferred immediately by pipette into a sterile cryovial. All samples from an individual steer were placed in separate sealed waterproof bags and placed in an ice cooler prior to transfer to −80°C storage within 4 h. Sampling and transport of materials was performed separately for BVDV and BoHV-1 groups. Strict biosecurity protocols were followed with full change out of personal protective equipment and order of entry for sampling, with collections occurring in Group 2 (BoHV-1) prior to Group 1 (BVDV) BoHV-1.

Throughout the study, animals were visually inspected daily for clinical signs of illness. Viral inoculations were expected to cause subclinical to mild clinical signs, including mild fever, upper respiratory signs (clear nasal discharge), and reduced appetite. Animals were inspected daily by animal health research personnel and were examined by veterinary staff on dates of collection −5, −4, −3, −2, −1, 0, 1, 2, 3, 4, 5, 6, 7, 8, 9, 10, 14, 21, and 28 in conjunction with collection of blood samples for virus isolation (VI). On days 0 and 28 blood was collected for virus neutralization (VN). Steers were allowed full recovery and maintained within the AHR herd following the study.

### Virological Assays and Cultures

#### Culture

Growth of BVDV for culture-based training aid development contained 1 x 10^6^ and 1 x 10^5^ cell culture of BVDV-1b AU526 (BVDV group) and BoHV-1 Colorado (Cooper) strain containing 1 x 10^7^ in minimal essential medium (MEM). Culture preparations were made using previously described methods (10).

#### Virus Isolation

Detection of BVDV was performed in buffy coat cells from whole blood samples of all cattle (BVDV and BoHV-1 groups) through co-cultivation with Madin-Darby bovine kidney (MDBK) cells in adaptation of previously described methods (23). Briefly, the buffy coat was reconstituted to 1mL total volume in MEM with 10% EQS (media) and layered over cells that had been seeded 24 h earlier in a 24-well plate. Following three freeze-thaw cycles to release intracellular material, lysates from this procedure were incubated for 72 h on MDBK cells and assayed in triplicate by an immunoperoxidase monolayer assay using the BVDV-specific monoclonal antibodies D89 and 20.10.6.

#### Virus Neutralization

Sera were separated from clotted blood following collection, heat inactivated at 57°C for 30 min, and stored at −80°C until analysis. A standard virus neutralization microtiter assay was used for the detection and quantification of BVDV antibodies in sera of all cattle, as previously described (24). Sera were tested for neutralizing antibodies using the BVDV cytopathic strain NADL. Testing of sera for antibodies against BoHV-1 (BoHV-1 group) was performed using the BoHV-1 Colorado strain as previously described (25).

### Training Aid Development

The POCR training aids were prepared for use in biological detection using a method similar to those previously described for explosives odor capture (22, 26) with biological target-specific modifications for sterilization, patent pending (27). Odor profiles were “charged” onto the polymer material in a biosafety hood using standard laboratory clean technique. This included wearing disposable nitrile gloves and aliquoting materials with sterile disposable pipette tips onto clean glass petri dishes for charging. This charging involved placing the training aids in proximity to, but not in direct contact with, raw materials to ad/bsorb VOCs emitted by respective targets or distractors. The aids were removed after the charging process and placed through a rigorous two-step, high heat, high pressure sterilization process consistent with biosafety protocols for restricted agents (13). This procedure was utilized to conduct the experiment under the most stringent circumstances for rendering a POCR that has been exposed to a biological agent safe for training. The sterilization process was performed under these conditions to serve as model for use of protocols and materials relevant to restricted and emerging agents. Training aids for initial training and baseline performance were made with cultures and training aids for testing and probing were made with nasal, salivary, blood, fecal and urine samples collected from days +6 to +10 as it represented the peak infective window.

#### Contamination Risk Assessment

A set of culture POCR training aids were made for contamination risk assessment. The POCR training aids were “charged” fresh in identical fashion to the training aids used in canine trials. To represent the highest level of risk, aids were sampled for possible surface contamination pre-sterilization. Virus was propagated from original stock culture. Two swabs were moistened for each plate, one with 1 ml PBS in a collection tube and the second with 1 ml media in a collection tube. Each swab was used to sample the entire surface of the POCR and placed into its respective tube resulting in 20 samples for BVDV (10 for each phosphate-buffered saline (PBS) and media, respectively) and 20 samples for BoHV-1 (10 for each PBS and media, respectively). Subsequently, 500 microliters of each tube were used for RNA/DNA extraction to perform qPCR detection, adapted from previously described methods (23). The assay utilized in this study involved use of a probe rather than SYBR green and using the QuantaBIO qScript XLT master mix. An additional 500 microliters were placed in −80°C for reserve pending positive VI testing follow up.

### Canines

#### Subjects

Six Labrador retrievers (2 M/4 F) between 1 to 5 years old (mean age: 2.35) from the Auburn University College of Veterinary Medicine (AUCVM) Canine Performance Sciences (CPS) detection dog program participated in this study. Dogs were housed in individual indoor/outdoor runs within the kennel complex at the AUCVM. The dogs were raised through the same breeding program and had similar puppy development and varying odor detection training experience prior to placement on the study. All dogs selected had no prior experience with biological or medical detection.

#### Training and Generalization Testing

**Figure 1:**
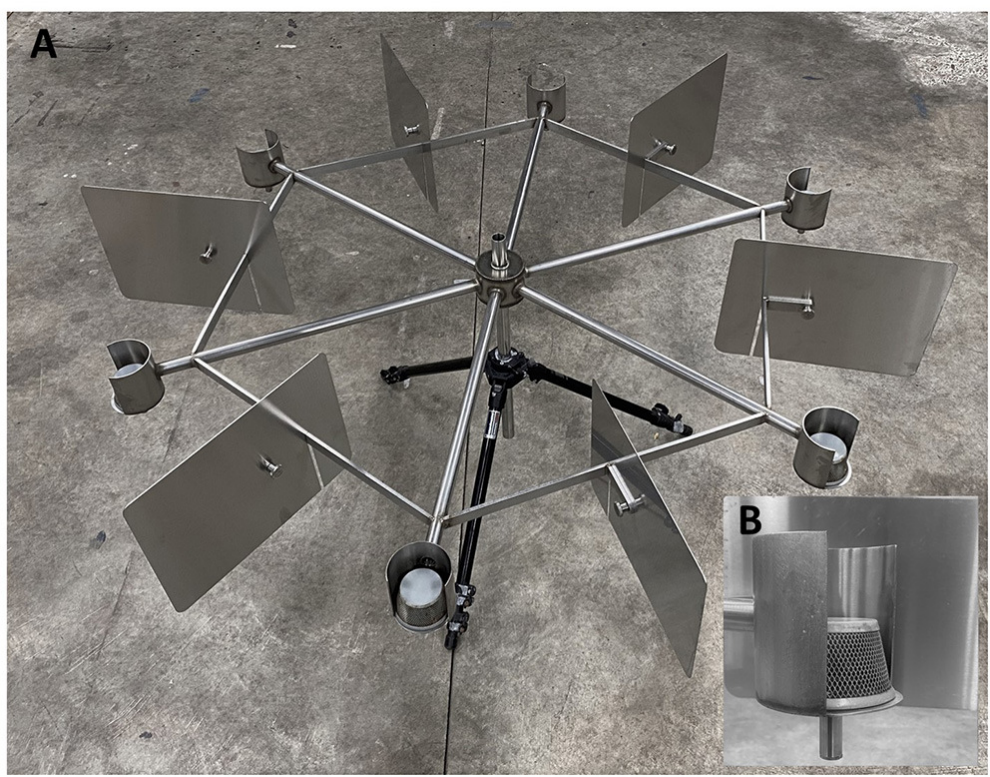
Testing Wheel Image: **(A)** Stainless-steel testing wheel with 6 radial arms and sample divider plates between positions; **(B)** Walled (3/4 circumference) cups and wire basket for sample presentation.

Training and testing occurred in a 4 x 4 m dedicated biosafety (BSL 2) training room that was climate- and humidity-controlled. In the center of the room was a stainless-steel scent wheel (1.31 m in diameter) with six arms (Figure 1A). A stainless-steel cup attached to the end of each arm held samples for presentation (9.53 cm in diameter) (Figure 1B). Upon placement of a sample (i.e., POCR), the cup was covered with a wire mesh basket to allow odor sampling while preventing physical contact between the dog and the substance (Figure 1B). Dogs were familiarized on the task of performing a wheel search prior to start of study.

**Figure 2:**
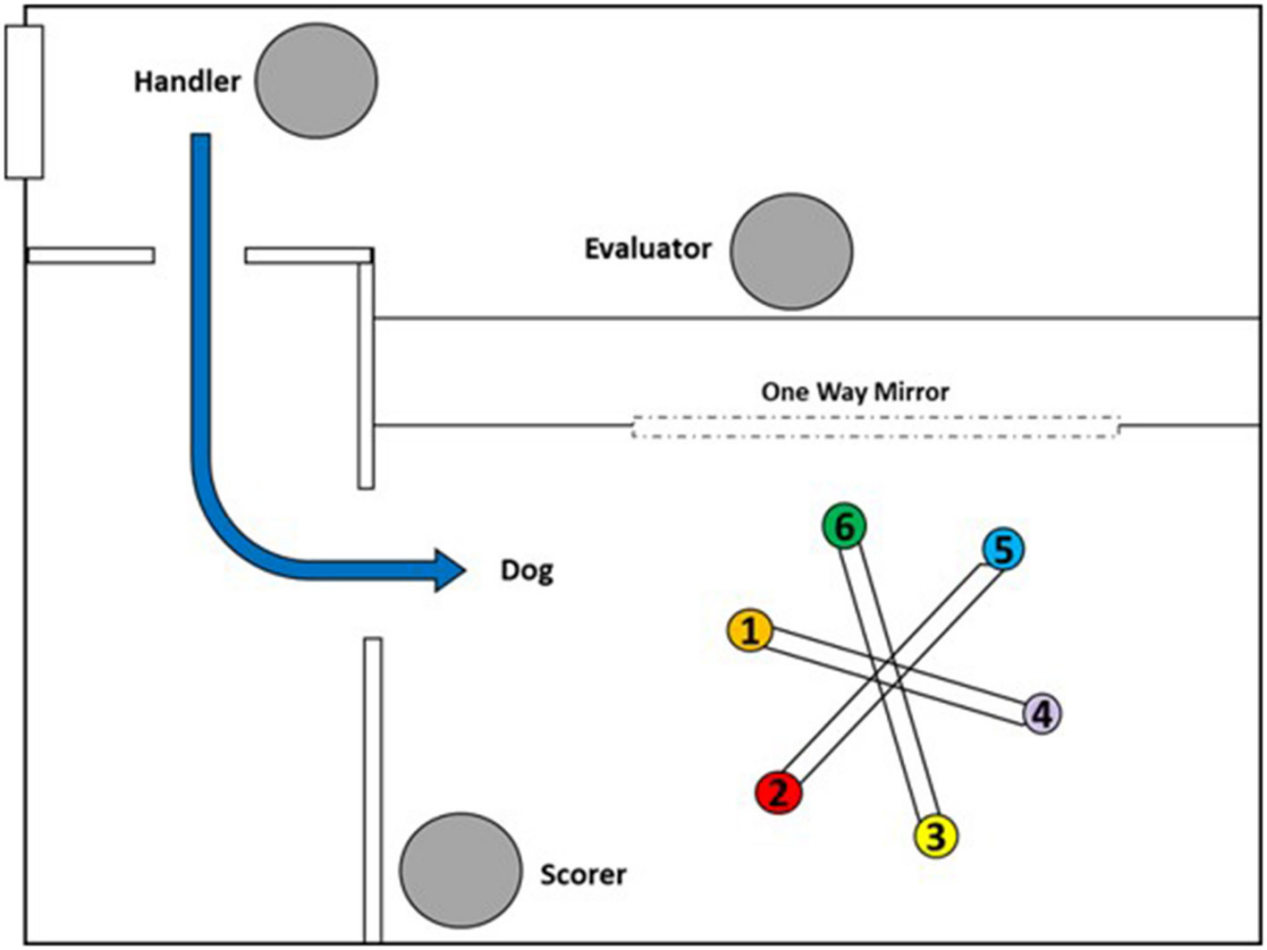
Room layout for wheel testing. Scorer is unaware of trial set-up and blind to target placement or trial type and unable to see the moderator behind the one-way mirror. Dog is released from the handler to enter the wheel room off-leash and search independently.

Test sessions were conducted single blind, with the dogs trained to work off leash. The handler always remained outside of the room out of the dog's sight (Figure 2). At the start of each trial, the experimenter placed the samples on the wheel and then exited the area, remaining inside a control room adjacent to the wheel room for the duration of the trial and viewed the dog through a one-way mirror. The handler then sent the dog into the room to sample the wheel. The experimenter signaled the outcome of each trial to the handler using hand signals only visible to the handler. If the dog made a correct indication (a sit response, operationally defined as full contact of the hindquarters on the ground for any duration in front of the target position (28) or searched the last position with no false alerts on trials with no target present, the experimenter signaled with a thumbs up and the handler recalled the dog and delivered a reward (play with a ball). If the dog searched all positions without making an indication when a target odor was present (false negative), the experimenter signaled with a thumbs down, and the handler called the dog out of the room without delivering a reward. The same call, “come” was used for calling the dog out of the room on all trials. If the dog responded at a position that did not contain a target (false alarm), the dog was ignored and allowed to continue searching the remaining positions. An observer who was blind to the presence and location of targets, positioned in the corner of the wheel room (Figure 2), scored whether and at which location dogs made a response.

Dogs were first trained to detect the odor of BVDV viral culture using the POCR training aid (see Table 1). Dogs were initially introduced to the odor using a standard odor discrimination line-up with stainless steel boxes within the same testing room along the straight corridor adjacent to the wheel, in which dogs were taught to associate the odor of the BVDV viral culture POCR with a reward (play with a ball) and to discriminate it from “blank” (i.e., uncharged) POCR. Training then progressed to the wheel scenario, culminating in a baseline session to serve as confirmation of the dogs' proficiency in detecting the trained target and to serve as a comparison with their proficiency in detecting the targets in the subsequent generalization tests. Number of training trials prior to baseline testing varied by individual dog based on chief instructor assessment of performance improvement and progression of odor learning. The baseline sessions were conducted over two consecutive days with 10 trials per session. Each session consisted of 6 target and 4 blank trials which were randomized across the session. The placement of targets was counterbalanced across the six positions, with distractors in all other positions. The non-target (“blank”) trials contained all distractors. Distractors included blank media whole, each media component, and a non-target viral culture (BoHV-1) (see Table 1).

**Table 1:** Culture POCR training and testing list. The list of target and distractors used in the training and testing of culture POCR.

**Category**	**Sample**
Target	BVDV Culture
Distractors	BoHV-1 Culture
	Media component: equine serum
	Media component: sodium bicarbonate
	Media component: antibiotic combination consisting of penicillin/ streptomycin/amphotericin (PSF)
	Media component: L-glutamine
	Media component: minimal essential media (MEM) with Earle's salts
	Media component: purified water
	Media Whole

Generalization tests (see Table 2) occurred the same as the baseline sessions with dogs completing only one session per day. The first probe odor (nasal sample POCR) was presented across three consecutive sessions consisting of 10 trials each to determine dogs' ability to generalize from viral culture POCR to sample POCR. Each trial presented 6 target and 4 blank runs randomized across the session. Next, dogs completed eight additional sessions, two for each sample type on POCR, in the following order: saliva, blood, urine, fecal, urine, saliva, fecal, blood. Responses to probe odors were reinforced like baseline trials to minimize disruption of performance. If deemed necessary by the chief instructor based on individual dog's task focus, search behavior and number of elapsed trials with no reward, a baseline trial with culture POCR was inserted to maintain motivation. The distractors selected included self-matched controls (pre-inoculation) for positive target samples in respective sample types, clinically similar viral positive samples (BoHV-1) and controls (pre-inoculation).

**Table 2:** Sample POCR generalization testing list. Each set of targets and distractors were used across each sample type.

**Category**	**Sample**
Target	BVDV Sample Animal (days +6 to +10)
Distractors	BoHV-1 Sample Animal (days +6 to +10)
	BVDV Self-Control (days−5 to−1)
	BoHV-1 Control (days−5 to−1)

*Six individual targets were selected each session at a 1:1 ratio with BoHV-1 sample and BVDV self-control as distractors*.

#### Controls

All targets and distractors and their holding containers were changed after each trial. Baskets, basket holders, scent wheel apparatus, and POCR devices/petri dishes were only handled using nitrile gloves and metal forceps to eliminate human scent (29). Baskets and petri dishes were sanitized with high heat after each use in a commercial dishwasher (up to 68°C). All targets and distractors were handled by the same person to eliminate the dogs' ability to identify a person-scent associated with the categories of samples. All personnel present donned gowns, gloves and goggles while conducting experiments. Distractor odors were present in all non-target positions to serve as negative controls for calculating specificity/false alarm rate. Each trial included self-matched controls (pre-inoculation) for each individual steer that would be presented during the trial (6 targets from days +6 to +10 and 6 self-matched controls from days−5 to−1). Days +6 to +10 were selected for animal sample aid presentations as it represented the peak infective window. Target samples were only presented once for each dog in a given sample type, no target samples were repeated across the trials for any given sample type for any individual dog.

#### Performance Scoring and Data Analysis

On each trial, dogs' responses were scored as a true positive (response to a position containing a target), false negative (no response to a position containing a target), false alarm (response to a position not containing a target), or true negative (no response to a position containing a distractor). Sensitivity for each target was calculated as total true positives out of total exposures to the target, averaged across all dogs across all sessions for that target. Specificity was calculated as total true negatives out of total positions searched, averaged across all dogs across all sessions for that target. Generalized linear mixed effects models (GLMMs) were used to analyze sensitivity and specificity as a function of the fixed factor of sample type (culture, nasal, saliva, blood, urine, and fecal). Analyses were performed in the R statistical program (Version 1.2.5033, RStudio). Data represent the mean (± SEM) unless otherwise noted. Additionally, we separately report total responses across dogs to the first presentation of each sample tested. Origin (Pro), Version 2021b. OriginLab Corporation, Northampton, MA, USA was used for receiver operator characteristics (ROC) curve analysis. A subset of videos (two randomly selected sessions for each sample type) was scored by an additional blind observer and total recorded dog sits and position checks were used to calculate inter-rater reliability, which was very good for total true positives (ICC = 0.98, *p* < 0.001), true negatives (ICC = 0.99, *p* < 0.001), false positives (ICC = 0.99, *p* < 0.001) and false negatives (ICC = 100, *p* < 0.001).

## Results

### Clinical Evaluations and Virological Assays

Following inoculation, cattle in the BoHV-1 group demonstrated clinical signs of infection at varying degrees across the course of infection to include hyperthermia and copious mucous to mucopurulent nasal discharge. Cattle in the BVDV group did not develop clinical signs of infection.

Virus neutralization results from all 20 BVDV-infected group 1 cattle demonstrated that all animals were successfully infected with BVDV, as indicated by a >4-fold increase from baseline on day 0 (1 ± 0 titer) to day +28 (62±19.6 titer) for BVDV. No measured increase for corresponding BoHV-1 results in group 1 cattle. The 10 BoHV-1 group 2 cattle demonstrated a >4-fold increase from baseline on day 0 (1 ± 0 titer) to day 28 (57.6 ± 9.29 titer) for BoHV-1 and no measured increase for corresponding BVDV results.

BVDV virus isolation results across all 20 BVDV group 1 cattle demonstrated viral detection in 16/20 individuals across a range from day +3 to day +10 with 2/20 individuals infected on day +3, 7/20 day +6, 13/20 day +7, 7/20 day +8, 2/20 day +9, 1/20 day +10 and 0/20 day +14.

### Canine Training and Generalization Testing

Dogs completed 143 training trials on average, across ~3 months, on the fixed sampling wheel. Baseline session confirmed that dogs were proficient in detecting the trained target (viral culture POCR), with 97.22% (± 2.78) sensitivity and 97.11% (± 1.94) specificity.

**Figure 3:**
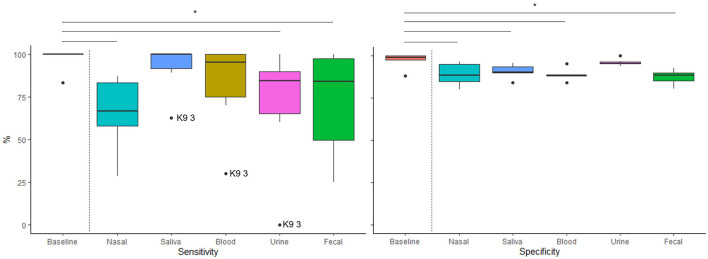
Distribution of canine (*n* = 6) sensitivity and specificity for each target (Culture, Nasal, Saliva, Blood, Urine and Feces). Horizontal lines inside boxes represent medians, boxes represent the interquartile range (IQR), and whiskers represent the range of values within 1.5 X IQR. Dots represent individual dogs that were determined outliers (either less than the first quartile - 1.5 X IQR or greater than the third quartile + 1.5 X IQR. Horizontal bars above the graph show significant differences in performance between baseline and sample tested.

Table 3 reports sensitivity and specificity for the baseline session with culture and each of the tested sample types. Sensitivity and specificity for each tested sample type as compared to baseline is shown in Figure 3. Sensitivities for nasal, urine, and fecal sample types were significantly lower than baseline sensitivity (GLMMs: *t* (25) = −2.88, *p* = 0.008; *t* (25) = −2.48, *p* = 0.020; *t* (25) = −2.27, *p* = 0.032, respectively), with no differences between saliva or blood compared to baseline (GLMMs: *t* (25) = −0.48, *p* = 0.634; *t* (25) = −1.41, *p* = 0.172, respectively). In addition, sensitivity for nasal samples was significantly lower than for saliva (GLMM: *t* (25) = −2.40, *p* = 0.024). There were no other significant differences in sensitivity between targets (*p* >.057). One dog (K9 3) represented the outlier across three sample types (see Figure 3).

**Table 3:** POCR testing results. Average (± SEM) sensitivity and specificity by dogs for each odor tested.

**Target**	**Sensitivity%**	**Specificity%**
Culture	97.22 (2.78)	97.11 (1.94)
Nasal	65.40 (8.98)	88.88 (2.72)
Saliva	91.90 (6.15)	90.64 (1.67)
Blood	81.67 (11.38)	88.61 (1.46)
Urine	69.81 (15.01)	96.00 (.89)
Fecal	72.13 (13.11)	87.36 (1.77)
Sample average	76.18 (10.93)	91.43 (1.68)

Specificities for nasal, saliva, blood, and fecal were significantly lower than baseline (GLMMs: *t* (25) = −3.61, *p* = 0.001; *t* (25) = −2.84, *p* = 0.009; *t* (25) = −3.73, *p* = 0.001; *t* (25) = −4.28, *p* < 0.001, respectively). However, there was no significant difference between urine and baseline (GLMM: *t*(25) = −0.47, *p* = 0.631). In addition, specificities for nasal, saliva, blood, and fecal were significantly lower than urine (GLMMs: *t* (25) = −3.12, *p* = 0.005; *t* (25) = −2.35, *p* = 0.027; *t* (25) = −3.24, *p* = 0.003; *t* (25) = −3.79, *p* < 0.001, respectively). Specificity across testing was above 90 % (*M* = 91.43, *SEM* = 1.68) (Table 3), indicating that dogs were discriminating the target virus from distractors.

Examining first-trial responses indicates that generalization varied by dog and across sample types. The probe presentation order in POCR was nasal presented across three consecutive sessions consisting of 10 trials each followed by eight sessions, two for each sample type, in the following order: saliva, blood, urine, fecal, urine, saliva, fecal, blood. For three sample types, first-trials responses were lower than the second trial responses (first trials nasal: 1/6, urine: 2/6, and fecal: 3/6; second trial nasal: 5/6, urine: 5/6, fecal: 4/6). In the other two sample types, all dogs generalized with high proficiency on first and second trials (first trial saliva: 5/6 and blood: 5/6; second trial saliva: 4/6, blood: 4/6).

**Figure 4:**
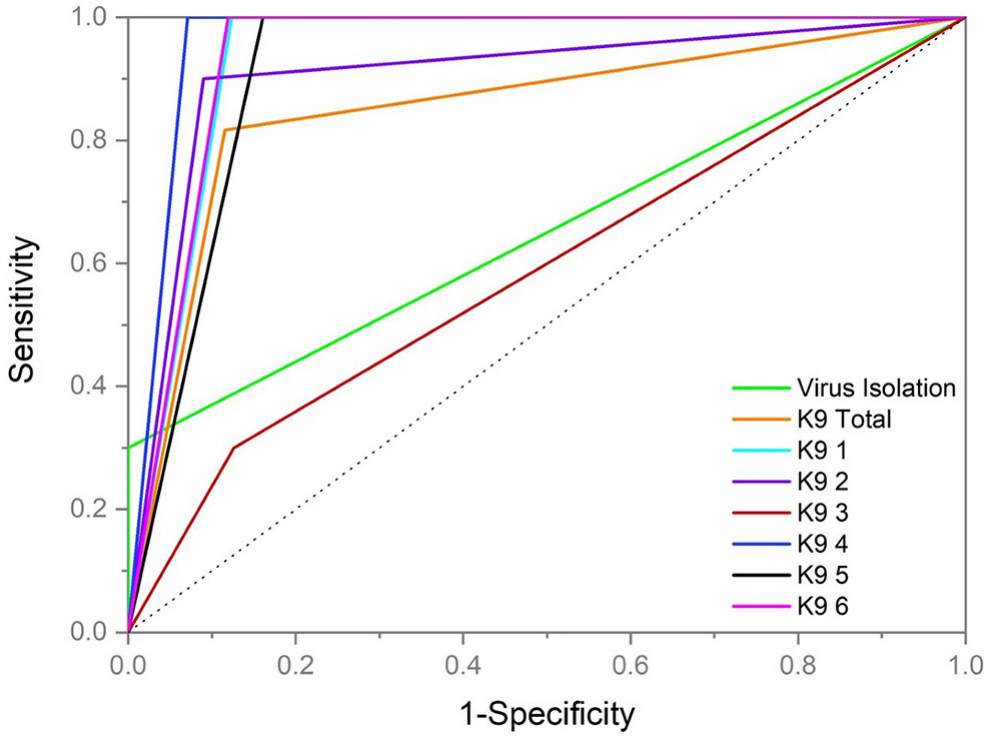
Graph of Receiver Operator Characteristic (ROC) curve representing all dogs (*n* = 6) as a test modality (K9 Total), each individual dog (K9 1, K9 2, K9 3, K9 4, K9 5, and K9 6) and virus isolation monitoring. K9 results represent blood sample testing during peak infective window samples of positive day +6 through +10 and corresponding trial control samples examined by the dogs. Virus isolation results represent all blood samples for virus isolation testing during peak infective window samples of positive day +6 through +10 and control samples. The dotted reference line runs through the center representing a diagnostic performance no better than chance.

Sensitivity and specificity of detection by dogs and by VI testing is graphically represented separately by the ROC curves in Figure 4. The ROC curve is graphical representation of the diagnostic ability of a binary classifier system plotting the true positive rate (sensitivity) in a function of the false positive rate (100-Specificity) and is a tool used for medical diagnostic test evaluation (30). Overall performance in blood POCR for each dog demonstrated K9 3 to have a lower area under the curve on the ROC analysis than VI in the cattle samples tested, while the remaining five dogs had a higher area under the curve than VI (Figure 4). All test curves demonstrated performance better than chance with respect to reference (dotted line Figure 4).

### Contamination Risk Assessment

Contamination testing was conducted on 20 representative POCR training aids, 10 each BVDV and BoHV-1. Each POCR swabbed twice, once with PBS and once with media. Swabs were analyzed using qPCR and results indicated four aids, 2 BVDV with media (C_q =_36.395 ± 0.007 SD) and 2 BoHV-1 with PBS (C_q__=_ 36.905 ± 0.049 SD), contained trace levels of genetic material. The four swabs representing the four training aids positive for trace levels of genetic material on qPCR were subsequently analyzed by virus isolation. All samples were negative on virus isolation for presence of live virus.

## Discussion

This study used a model virus (BVDV) to demonstrate the utility of a polymer-based training aid to capture a biological agent-based odor profile for use in training and testing for sample-based odors. The results indicate that BSL3 decontamination and odor absorption strategies for the POCR training aid technology hold application toward biological detection and support the perceptive presence of a unique biological agent-based odor profile for BVDV distinguishable from another representative clinically similar virus (BoHV-1) across multiple sample types.

The unique biological agent-based odor profile perceived by trained detection canines in culture-based aids were shown to be recognizable, with varying rates of generalization, across the five sample types. All dogs met a high level of performance of over 97% sensitivity and specificity in the culture prior to testing with the samples. Detection rates to the tested samples ranged from a low of 65.40% sensitivity in nasal samples and 87.36% specificity in feces to a high of 91.90% sensitivity in saliva and 96% specificity in urine. These results indicate a moderate to high rate of confidence in the presence of a unique odor associated with BVDV culture that relatively preserved in aid development post-autoclave procedures. This unique odor is also shared across sample types (nasal, saliva, blood, urine, and feces). Further, that unique odor is also a commonality shared across separate BVDV positive individuals as each steer's positive sample type was presented during testing only once for each dog and stringent controls were used to prevent use of individual animal cues by the dog for target recognition.

Across all dogs, one outlier (K9 3) represented the highest contribution to variance. Individual variability in generalization by dogs has been reported (21, 31), which could be the result of numerous uncontrolled or unknown factors such as training history, age, or temperament. With a small sample size, this variability supports the need for further research to explore factors contributing to individual differences in generalization. However, these differences may underscore the exigent requirements of detection dogs to perform the complex detection tasks of biological targets across multiple contexts, which may result in a narrower criterion for dog selection in this field.

Generalization testing performed with culture-trained dogs across the five sample types was lowest to nasal samples (65%), which could be due to several unknown factors such as collected sample odor retention rates and sample type features (e.g., mucus), or a procedural factor such as testing order given that nasal samples were the first to be tested following culture training. The improvement in the second trial for nasal samples likely represents a challenge in transition of context for generalization from a culture-based context to a sample-based context. The change in target context, from culture to cattle sample, represents a variation to the presented odor profile and introduces additional background odors. With individual sample-based training, detection dogs have shown an ability to discriminate positive individuals from negative individuals based on individual scent vs. condition-associated odor, such as disease or infective state (32). To control for this, dogs were tested only once with any positive individual in a given sample type with no repeat exposures, Additionally, self-matched negative controls of the positive targets (i.e., pre-inoculation) were used as distractors within the same session. Thus, the use of self-controls and no repeat exposures suggests that this improvement is not attributable to individual-based sample learning.

The subsequent sample type tested, saliva, demonstrated that the relatively non-invasive sampling of saliva yielded high generalization on first trial responses across the six dogs at 5/6, which may indicate that, after an initial context generalization occurs from the culture-based to sample-based context, the subsequent rate of generalization for additional sample types improves. Overall, between the two non-consecutive sessions, dogs showed no significant differences in sensitivity on saliva from culture-baseline even with the outlier of K9 3. Additionally, the next tested sample (blood) yielded high generalization on first trial responses across the six dogs at 5/6. Overall, between the two non-consecutive sessions, dogs maintained high sensitivity not significantly different from baseline, but with a wider overall range across individual dogs with no single significant outlier compared to saliva. However, upon presentation of urine and feces the rate of generalization during the first session dropped, though remained higher than the initial seen with nasal. The complex odor matrices represented in urine and feces increase the amount of background odor noise anticipated due to representing the two primary elimination pathways for bodily waste products. This likely presents a larger initial challenge to generalization that may improve on subsequent exposures due to context generalization.

Virus neutralization (VN) demonstrated a successful inoculation across all cattle with BVDV in group 1 and the virus isolation (VI) monitoring across multiple days demonstrated peak period of viral shed at days +6, +7 and +8. Using virus isolation as a screening tool was highly specific (100% ±0) with lower sensitivity during the expected peak window day +6 to +10 (30% ± 1). Though not a goal of this study to compare diagnostic capabilities the results of the virus isolation tests, which currently represent the “gold standard” in screening techniques for cattle, indicate that the dogs were more sensitive to positive cattle samples than VI. In the ROC curve analysis performed to evaluate these differences using the same sample type (blood), the dogs' results indicated an overall higher sensitivity (81.67% ± 11.38) but lower specificity (88.61% ± 1.46). Individual dog performance varied with one dog, K93, demonstrating an overall lower area under the curve value than virus isolation. The cattle results were VN-positive in all 20 individuals confirming infection, but only VI-positive in 16/20 individuals on at least 1 day leaving 4 individuals VI-negative across all testing days. The total dog screening results across those same 4/20 VN-positive but VI-negative cattle were 64.17% (±11.16) in sensitivity. These data appear to suggest that the presence of virus detectable by traditional means (i.e., VI) in a sample is not necessary for odor recognition and the metabolic processes that occur due to infection, non-intact virus and/or genetic material are more suggestive to result in the unique biological agent-based odor profile for BVDV distinct from BoHV-1. Use of culture-based training advancing to sample testing with successful generalization in dogs is also suggestive that a systemic response to infection is not necessary for presentation of a unique biological agent-based odor profile in BVDV.

A measure of quality assurance to monitor for possible contamination of training aid materials despite strict indirect charging conditions with clean technique was performed using the highest potential state of risk: a set of fresh unautoclaved POCRs for each virus used, BVDV and BoHV-1. The results of this surveillance showed rare, low-level contamination of genetic material present with no live virus detected on any samples. These results support the overall need for the rigorous sterilization process for safe processing and fielding of training aids to operational canine teams.

This study indicates a capability for safely training detection canines in the context of restricted and hazardous biological targets using the POCR training aid. Future studies should evaluate the best training methods for generalization from POCR to live animal and field-based testing. Using a controlled prospective study to evaluate windows of detection (early incubation period vs. non-infective recovery) beyond the peak infective period will be useful in establishing the limits of this capability. In addition, the analyses of the VOCs may reveal an odor fingerprint that is biological agent-specific which could be applied toward electronic sensing and screening modalities. The characteristics of the odors emitted by the biological targets in this study are unknown; therefore, extrapolating results with targets in this study to other biological targets should be done with caution. Any specific target biological agent needs to be tested in a manner that illustrates operational effectiveness. The odors that dogs use to interpret the viral cultures and which odors of the viral culture are captured and delivered by POCR are currently unknown. The practical utility of the detection dogs' capability demonstrated in this study should be further investigated through operational testing.

## Data Availability Statement

The raw data supporting the conclusions of this article will be made available by the authors, without undue reservation.

## Ethics Statement

The animal study was reviewed and approved by Auburn University College of Veterinary Medicine Institutional Animal Care and Use Committee.

## Author Contributions

CA, TP, and MS: conceptualization and methodology. TF, TP, and MS: data collection. SS, SK, LL, and MS: analysis. CA, SK, and MS: writing—original draft preparation. CA, LW, PW, and MS supervision. All authors contributed to review and editing and approved the final version of the manuscript.

## Funding

This research was funded by FBI Weapons of Mass Destruction Directorate, Emerging Threats and Technologies Unit, Chemical Biological Advanced Threat Detection, grant number FBI-15F06718P0002696-BCU-D3 and FBI-15F06718P0002696-BCU-L2.

## Conflict of Interest

CA, TP, TF, and LW are represented on the patent, pending (TRAINING AID Patent Pending 2020 338518: 47-19 US). The remaining authors declare that the research was conducted in the absence of any commercial or financial relationships that could be construed as a potential conflict of interest.

## Publisher's Note

All claims expressed in this article are solely those of the authors and do not necessarily represent those of their affiliated organizations, or those of the publisher, the editors and the reviewers. Any product that may be evaluated in this article, or claim that may be made by its manufacturer, is not guaranteed or endorsed by the publisher.
